# Transumbilical laparoendoscopic single-site radical trachelectomy with uterine arteries’ ascending branches preservation for early-stage cervical cancer

**DOI:** 10.1136/ijgc-2022-003887

**Published:** 2022-12-16

**Authors:** Yu Chen, Ying Zheng, Qiao Wang, Siyu Dai

**Affiliations:** 1 Department of Gynecologic Oncology, West China Second University Hospital, Chengdu, Sichuan, China; 2 Key Laboratory of Birth Defects and Related Diseases of Women and Children (Sichuan University), Ministry of Education, Chengdu, China

**Keywords:** Cervical Cancer, Gynecologic Surgical Procedures, Sentinel Lymph Node, Laparoscopes

Cervical cancer is the fourth-leading cause of cancer incidence and death among females according to Global Cancer Statistics 2018.^1^ With an increasing number of young women being diagnosed with cervical cancer, fertility-sparing surgery is widely demanded. According to 2022 National Comprehensive Cancer Network guidelines, radical trachelectomy and pelvic lymphadenectomy are indicated for patiens with stage IA2, stage IB1, and selected stage IB2 cervical cancers, meaning minimally invasive approach remains an option. The transumbilical laparoendoscopic single-site (TU-LESS) approach is now gaining in popularity for treating gynecologic diseases because of its convenient specimen extraction, better cosmesis, reduced post-operative pain, and shorter recovery period.^2^ To the best of our knowledge, this is the first case in which the whole laparoscopic procedure of radical trachelectomy through TU-LESS approach has been reported.

This video demonstrates the fertility-sparing surgery procedure containing radical trachelectomy and pelvic lymphadenectomy with bilateral ascending branches of uterine arteries preservation. The patient was a 34-year-old woman diagnosed with International Federation of Gynecology and Obstetrics stage IB1 moderately differentiated squamous cervical cancer who expressed a strong desire for reproduction. After sufficient consultation, she decided to receive a fertility-sparing treatment protocol. This video highlights the feasibility of sufficiently dissecting paracervical structures and exposing uterine artery branches with complex procedures and no assistance via TU-LESS. We adopted a simplified uterine manipulator, which minimized the squeezing and injury to the cervix, to assist exposure of the surgical field. Resection of the cervix was accomplished transvaginally with a cold knife to ensure sufficient margin. Pathology examination of surgical margin, lymph node specimens, and lymphovascular invasion were negative. The umbilical incision was closed by Zheng’s anchor suturing technique to prevent incisional complications and improve cosmetics.^3^ No disease recurrence or surgical complications were detected in the 6 months of follow-up.

Minimally invasive surgery is safe and effective for fertility-sparing without compromising oncologic outcomes, especially in patients with a tumor size <2 cm.^4^ TU-LESS is a feasible option for delicate dissection in radical trachelectomy and can achieve minor incision and faster recovery. However, owing to the limited operational space and absence of assistance in TU-LESS, this type of surgery requires an extensive learning curve before performance.

**Figure 1:**
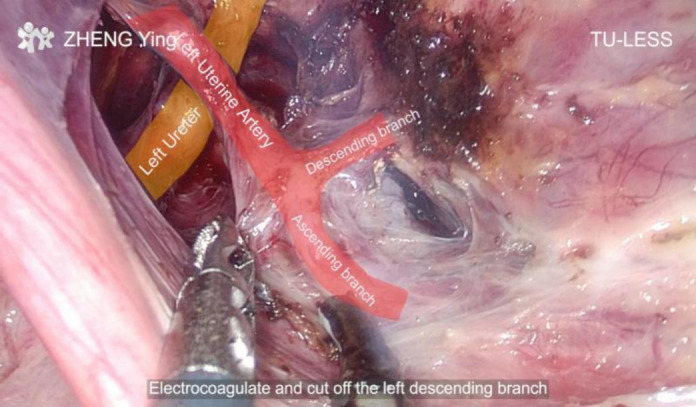
Exposure of uterine artery branches.

**Figure 2:**
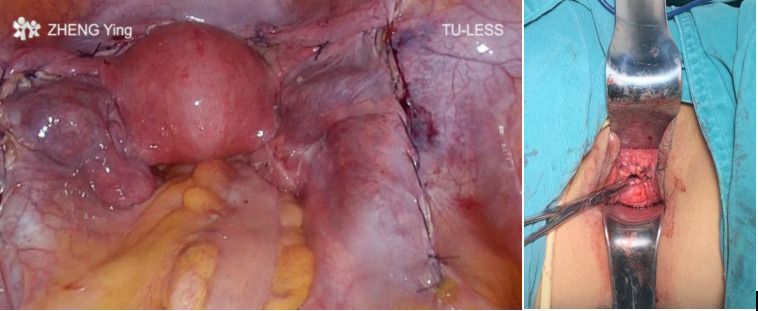
Inside and outside view after radical trachelectomy.

## Data Availability

All data relevant to the study are included in the article and video.
